# A Z-Linked E3 Ubiquitin Ligase *Cs-rchy1* Is Involved in Gametogenesis in Chinese Tongue Sole, *Cynoglossus semilaevis*

**DOI:** 10.3390/ani11113265

**Published:** 2021-11-15

**Authors:** Yuxuan Sun, Ying Zhu, Peng Cheng, Mengqian Zhang, Na Wang, Zhongkai Cui, Min Wei, Wenteng Xu

**Affiliations:** 1 Laboratory for Marine Fisheries Science and Food Production Processes, Pilot National Laboratory for Marine Science and Technology, Yellow Sea Fisheries Research Institute, Chinese Academy of Fishery Sciences (CAFS), Qingdao 266071, China; sunyx0317@163.com (Y.S.); zhuying@qau.edu.cn (Y.Z.); m190100126@st.shou.edu.cn (P.C.); mengqian154273@163.com (M.Z.); wangna@ysfri.ac.cn (N.W.); cuizk3@foxmail.com (Z.C.); 2 Jiangsu Key Laboratory of Marine Biotechnology, Jiangsu Ocean University, Lianyungang 222005, China; weimin@jou.edu.cn; 3 School of Marine Science and Engineering, Qingdao Agricultural University, Qingdao 266237, China

**Keywords:** *Cynoglossus semilaevis*, *Cs-rchy1*, ubiquitin ligases, expression pattern, RNA interference

## Abstract

**Simple Summary:**

The sexual growth dimorphism prevails in animals and this phenomenon is even more obvious in marine fish, so understanding the mechanism of gonadal development and gametogenesis is of great importance for sex control, thus increased productivity in aquaculture. In mammal, ubiquitin ligase plays a versatile role in gonadal development and spermatogenesis, whereas its function in fish is little reported. Using *Cynoglossus semilaevis* (one-year-old female individual usually grows 2–4 times bigger than male) as the fish model, a Z-chromosome linked ubiquitin ligase *neurl3* was previously identified and characterized, which suggested its involvement in spermatogenesis. However, in this study, characterization of another Z-chromosome linked ubiquitin ligase *Cs-rchy1* suggested it might function both in spermatogenesis and oogenesis, as well as the potential role in growth. These data may provide the genetic resource for gene editing or marker exploration in future.

**Abstract:**

Ubiquitin ligase (E3) plays a versatile role in gonadal development and spermatogenesis in mammals, while its function in fish is little reported. In this study, a Z-chromosome linked ubiquitin ligase *rchy1* in *C. semilaevis* (*Cs-rchy1*) was cloned and characterized. The full-length cDNA was composed of 1962 bp, including 551 bp 5′UTR, 736 bp 3′UTR, and 675 bp ORF encoding a 224-amino-acid (aa) protein. *Cs-rchy1* was examined among seven different tissues and found to be predominantly expressed in gonads. In testis, *Cs-rchy1* could be detected from 40 days post hatching (dph) until 3 years post hatching (yph), but there was a significant increase at 6 months post hatching (mph). In comparison, the expression levels in ovary were rather stable among different developmental stages. In situ hybridization showed that *Cs-rchy1* was mainly localized in germ cells, that is, spermatid and spermatozoa in testis and stage I, II and III oocytes in ovary. In vitro RNA interference found that *Cs-rchy1* knockdown resulted in the decline of *sox9* and *igf1* in ovarian cell line and down-regulation of *cyp19a* in the testicular cell line. These data suggested that *Cs-rchy1* might participate in gonadal differentiation and gametogenesis, via regulating steroid hormone synthesis.

## 1. Introduction

Ubiquitination is a fundamental process that controls protein homeostasis by adding ubiquitin to the protein, which will be subject to degradation. Ubiquitination involves the coordination by three classes of enzymes, ubiquitin-activating enzyme (E1), ubiquitin conjugating enzyme (E2), and ubiquitin-protein ligase (E3) [1]. E1 activates and binds ubiquitin molecules by hydrolyzing ATP, ubiquitin was sequentially transferred to E2 and E3, finally to the substrate (target protein). Among the three enzymes, E3 is responsible for the recognition and binding of substrates, which determines the substrates specificity. E3 participates in various physiological processes, such as growth morphogenesis and sexual development [2,3]. Recently, accumulating reports have suggested E3 plays a role in reproduction and sexual differentiation in mammals. For example, data from mouse cell lines suggest that E3 participate in the positioning and the attachment of XY body in pachytene spermatocytes [2] and an E3 ligase *rnf31* cooperates with DAX-1 to regulate steroidogenic pathways [4]. Since Rajapurohitam et al. found that the ubiquitination level of rat testis was significantly higher than that of other tissues [5], a number of in vivo studies have focused on the role of E3 in spermatogenesis. In mice, E3s (ubr2, Siah1a and Cullin 4A) functioned on meiotic stage of spermatogenesis, and their deletion would result in the stagnation of germ cell differentiation, reduction of sperm number and motility [6,7,8], while other E3 (rnf133, TMF/ara160, herc4) played a role in spermiogenesis, the mutation of which mainly led to abnormalities in sperm morphology and motility [9,10,11].

In fish, ubiquitination also plays an important role in sexual differentiation and spermatogenesis. In eel, the ubiquitination level is increased during gonadal transformation and gametogenesis. Based on this, a ubiquitin carboxyl terminal hydrolase UCH-L1 (a type of deubiquitinase) was found to be highly expressed in these processes and may play an important regulatory role [12]. In rainbow trout, histone ubiquitination strictly regulated the replacement of protamine in spermatogenesis [13]. Using the subtractive library screening method, 32 candidate genes were related to spermatogenesis in dogfish, including ubiquitin carboxyl terminal hydrolase uch-l3 [14]. Similarly, over 400 genes were found to be related to spermatogenesis in Senegalese sole, including several ubiquitination-related genes, such as E1 and ubiquitin carboxyl terminal hydrolase uch, suggesting the important role of ubiquitination in spermatogenesis [15]. However, the study in fish mainly focused on gene identification and the report of their function and their regulatory role was rare. Chinese tongue sole (*Cynoglossus semilaevis*) is an economically important aquaculture species that is widely cultured in China. Tongue sole exhibits obvious sexual growth dimorphism, with female individuals being 2–4 times bigger than males, and so sex control techniques would benefit the industry. In this study, we have identified a Z-linked E3 ubiquitin ligase *rchy1* and performed the characterization by sequence analysis, expression profile, cellular localization and in vitro RNA interference. The data have suggested its role in sex differentiation and gametogenesis, which would provide new genetic resources for exploring sex control techniques.

## 2. Materials and Methods

### 2.1. Ethics Approval

The study was performed under the inspection of the committee at the Yellow Sea Fisheries Research Institute (Approval number, YSFRI-2021018). MS222 was used for anesthesia to minimize fish suffering (solubilized in seawater, final concentration 20 mg/L, fish was treated for 5 min) during experimental procedure.

### 2.2. Fish and Tissue Collection

Chinese tongue sole was obtained from Haiyang High-Tech Experimental Base (Haiyang, Shandong Province, China). Tissue samples from mature fish (3-year-old male and female), including spleen, heart, intestine, brain, kidney, liver, and gonads, were all collected, immediately frozen in liquid nitrogen and then stored at −80 °C. Gonads from tongue sole at different developmental stages, including 40, 60 and 90 days post hatching (dph), 6 months post hatching (mph), 1.5 and 3 years post hatching (yph), were also picked up. Gonad samples were either placed in liquid nitrogen and then preserved at −80 °C for RNA extraction or incubated in 4% (*w*/*v*) paraformaldehyde fixative, which would be used for in situ hybridization (ISH). Tail fins were cut and stored in absolute ethanol for DNA extraction and genetic sex determination.

Genetic sex was determined according to previously described methods [16]. In brief, genomic DNA extracted from fin sample was used as a template and PCR amplification was performed using a sex-specific primer combination (scaffold68-2F and scaffold68-2R) (Table 1). Only 169 bp band was observed for male sample while 134 and 169 bp bands were seen for female samples.

### 2.3. Sequence Analysis and Alignment

*Cs-rchy1* (accession number 103397453) is annotated on NCBI (https://www.ncbi.nlm.nih.gov/gene/103397453 (accessed on 14 June 2021). Open reading frame (ORF) and the encoded polypeptide was anlayzed with DNASTAR 7.10 (http://www.dnastar.com/ (accessed on 21 July 2021). The molecular weight (Mw) and theoretical pI were calculated on http://web.expasy.org/ (accessed on 21 July 2021). The conserved domain was predicted on Simple Modular Architecture Research Tool (SMART) (http://smart.embl-heidelberg.de/ (accessed on 21 July 2021). Phylogenetic tree was conducted using MEGA 6.0 by employing neighbor-joining method.

### 2.4. cDNA Synthesis and Quantitative Real-Time PCR (qPCR) Analysis

Total RNA (800 μg) was isolated using TRIzol reagents (Invitrogen, Carlsbad, CA, USA) according to the manufacturer’s instructions. The reverse transcription was conducted using a PrimeScript™ RT reagent kit (TaKaRa, Otsu, Japan). qPCR analysis was performed using TAKARA TB Green Premix Ex Taq II (TaKaRa) with 7500 ABI real time PCR machine (Applied Biosystems, Foster City, CA, USA). In brief, a volume of 20 μL reaction system was prepared, containing 10.0 μL 2× SYBR Premix, 0.4 μL of each sense and anti-sense primer, 0.4 μL ROX Dye II, 1.0 μL cDNA, and 7.8 μL ddH_2_O. PCR program was as follows: 30 s at 95 °C, 40 cycles of 95 °C for 5 s and 60 °C for 34 s, then default program of melting curve. β-actin was used as internal reference (Dong et al. 2016). The results were analyzed and the data were expressed as mean ± S.D. Differences (*p* < 0.05) were defined as significant. Primers used in this study are shown in Table 1.

### 2.5. Cellular Localization of Cs-rchy1 mRNA in Gonads

To examine *Cs-rchy1* expression patterns in the gonad, in situ hybridization (ISH) was carried out as previously described (Chen et al., 2014). In brief, the primer pairs *rchy1* probe F and probe R were designed (Table 1) to amplify 213 bp fragment and inserted into pBluescript II SK (+). The resultant recombinant plasmid was linearized with *Eco*R V and *Pst* I, transcribed by T3 or T7 RNA polymerase to generated digoxin (DIG) labeled sense or antisense RNA probes. Gonadal sample slices from 1.5 yph fish was subject to deparaffination and then incubated with probes (final concentration 0.2 μg/mL) at 50 °C overnight. Sample slices were blocked for 4 h (10% goat serum, 150 mM NaCl, 100 mM maleic acid, adjust to pH 7.5) at room temperature before anti-DIG-antibodies (Roche) were added for overnight incubation. The signal was finally developed using nitroblue tetrazolium/5-bromo-4-chloro-3-indolyl phosphate (Roche, Mannheim, Germany) and photos were capture by Nikon EClIPSE 80i microscopy. Gonadal from three male and three female were used for analysis and 3–4 slides were examined for each individual.

### 2.6. siRNA-Mediated Interference of Cs-rchy1 in Gonadal Cell Lines

Two specific small interfering RNAs (siRNAs), *rchy1-siRNA* and *rchy1-siRNA-i*, and a nonspecific siRNA as negative control (NC) were designed and synthesized by Sangon Co., Ltd. (Shanghai, China). The testicular and ovarian cells used for the RNAi were performed according to the procedure [17] using previously established testicular and ovarian cell lines (derived from testis and ovary and predominantly composed of somatic cells) [18,19]. Compared to *rchy1-siRNA-i*, *rchy1-siRNA* showed higher silencing efficiency (data not shown) and was employed for further experiment. Three replicates were conducted for *rchy1-siRNA* and NC groups. After transfection for 48 h, total RNA was extracted and reverse-transcribed according to above-mentioned methods. The expression profile of figla_tv1 (KT966740.1), *sox9* (NM_001294243.1), *sox-9-A* (XM_008315177.3), *cyp19a* (NM_001294183.1), insulin-like growth factor (*igf1*, NM_001294198.1) was measured by qPCR (primer sequences listed in Table 1).

## 3. Results

### 3.1. Cloning and Characteristics of CS-rchy1

**Figure 1:**
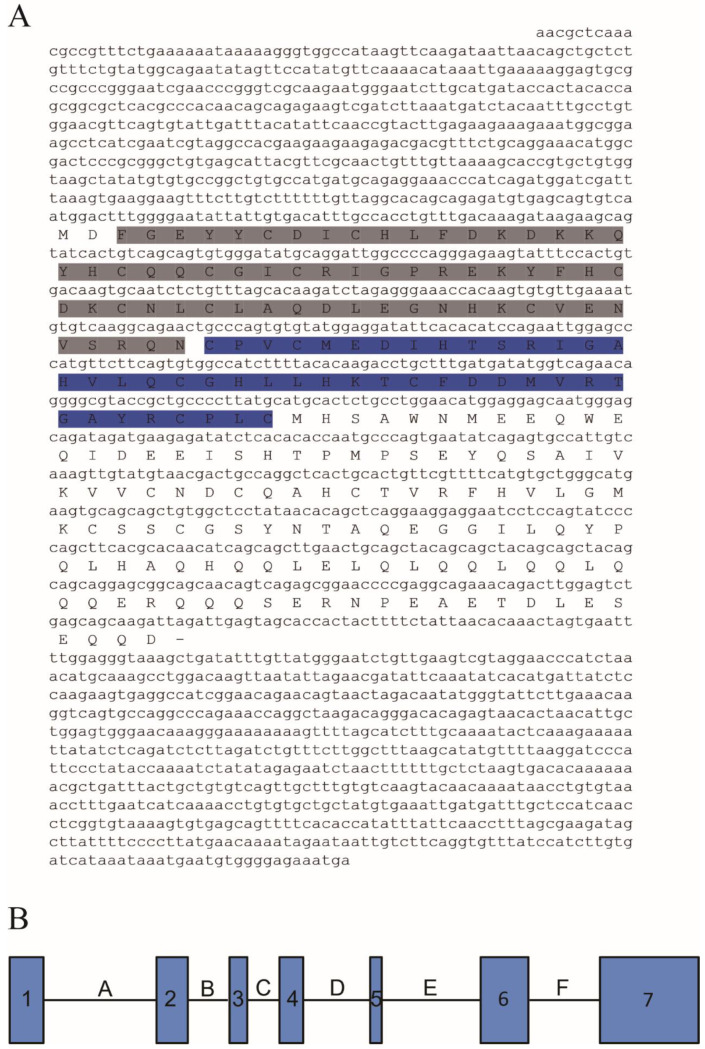
(**A**) The full-length cDNA and deduced amino acid sequence of *Cs-rchy1*. Amino acids in grey indicated the zinc finger CTCHY-type profile and amino acids in blue indicated the zinc finger RING-type profile. (**B**) the schematic representation of *Cs-rchy1* genomic structure. The exons were shown by 1–7 and only covered the CDS region (The length of exon 1–7: 86, 80, 45, 61, 29, 122, 252 bp). The introns were shown by uppercase A–F (The length of introns A–F: 291, 105, 81, 168, 254, 180 bp).

**Figure 2:**
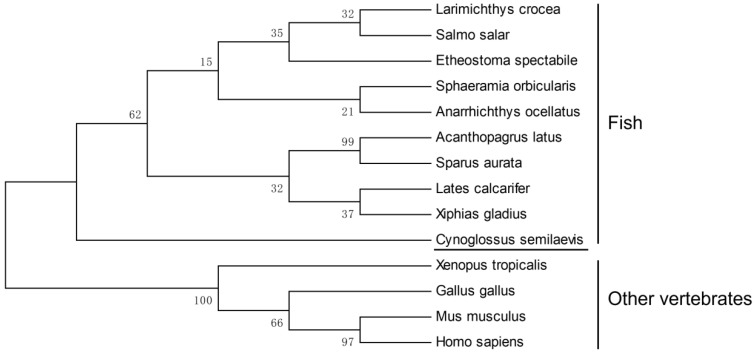
Phylogenetic analysis of RCHY1 from *C. semilaevis* and other vertebrates. Numbers at nodes represent NJ bootstrap values. The GenBank accession numbers of the amino acid sequences were as follows: *Cynoglossus semilaevis*, XP_024908814.1; *Acanthopagrus latus*, XP_036955304.1; *Sparus aurata*, XP_030271559.1; *Sphaeramia orbicularis*, XP_030000139.1; *Etheostoma spectabile*, XP_032372704.1; *Anarrhichthys ocellatus*, XP_031705216.1; *Lates calcarifer*, XP_018524733.1; *Xiphias gladius*, XP_040010866.1; *Larimichthys crocea*, TMS03383.1; *Salmo salar*, XP_014026963.1; *Xenopus tropicalis*, NP_001011487.1; *Gallus gallus*, NP_001074357.1; *Mus musculus*, NP_080833.1; *Homo sapiens*, NP_056251.2.

As shown in Figure 1, the 1962 bp *Cs-rchy1* full-length cDNA was acquired including 551 bp 5′UTR, 675 bp ORF and 736 bp 3′UTR fragments (GenBank accession no. 103397453). The ORF contains encoded a 224-amino-acid (aa) protein with predicted molecular weight 26.02 kDa and isoelectric point 5.36. Based on conserved domain prediction, the protein composed Zinc finger CTCHY-type domain (aa 3–65) and Zinc finger RING-type domain (aa 66–108), both of which were located near the N-terminus. Upon the analysis of genomic sequence, we have found the ORF region was composed of 7 exons, which were separated by 6 introns. Phylogenetic analysis showed vertebrate RCHY1 were clustered into two groups, where Cs-RCHY1 and other fish RCHY1 formed one group, and other vertebrate RCHY1 formed the other group (Figure 2).

### 3.2. Tissue Expression Patterns of Cs-rchy1

**Figure 3:**
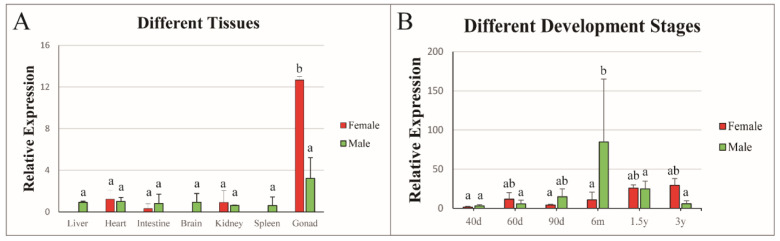
Relative expression of *Cs-rchy1* in different tissues (**A**) and different developmental stages (**B**). Values are indicated as means ± S.D (N = 3). The expression levels with the same letter are not significantly different (*p* < 0.05).

To determine the tissue distribution of *Cs-rchy1* in different tissues, qPCR was conducted using total RNA from seven different tissues of 3 yph female and male tongue sole. As shown in Figure 3A, *Cs-rchy1* could be detected in all tissues. It showed the highest expression in the gonads, while the expression level was much higher in the testis than in the ovary. In comparison, *Cs-rchy1* exhibited low expression in other tissues.

### 3.3. Expression Profile of Cs-rchy1 at Different Developmental Stages of Gonads

*Cs-rchy1* expression profile was examined in different stages of gonadal development (40, 60 and 90 dph, 6 mph, 1.5 and 3 ypp). As shown in Figure 3B, *Cs-rchy1* expression could be detected at all developmental stages. In the ovary, *Cs-rchy1* expression was rather stable along the developmental stages, although the level at 1.5 and 3 ypf was increased. In the testis, *Cs-rchy1* expression level increased gradually from 40, 60 and 90 dph, reached its peak level at 6mph, then declined at 1.5 and 3 yph.

### 3.4. Localization of Cs-rchy1 mRNA in Gonads

**Figure 4:**
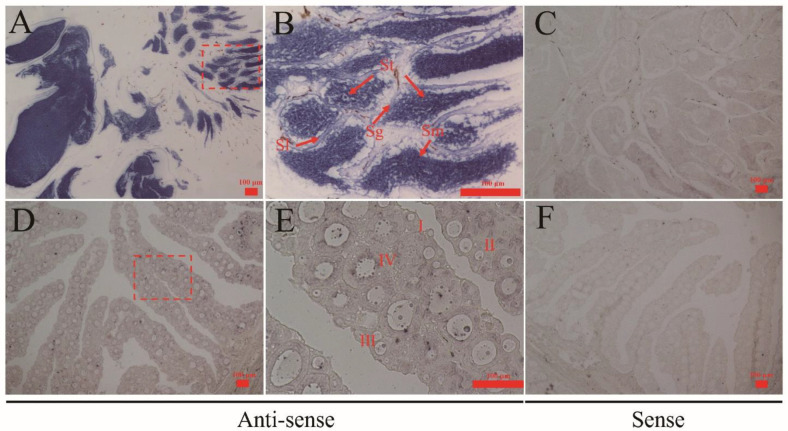
Cellular localization of *Cs-rchy1* in 1.5 yph gonads of *C. semilaevis*. The figure shows the testes (**A**–**C**) and ovaries (**D**–**F**). (**A**) testis labelled with *Cs-rchy1* antisense probes; (**B**) large magnification of red framed area in (**A**); (**C**) testis labelled with *Cs-rchy1* sense probes; (**D**) ovary labelled with *Cs-rchy1* antisense probes; (**E**) large magnification of red framed area in (**D**); (**F**) ovary labelled with *Cs-rchy1* sense probes. Sg: spermatogonia; St: spermatid; Sm: sperm; Sl: seminal lobule. Oocytes at different developmental stages are marked by I, II, III and IV. Scale bars: 100 μm.

To investigate the cellular localization of *Cs-rchy1* in gonads, ISH was performed in testis and ovary. As shown in Figure 4A,B, intense signals were located in both spermatids and sperm. In the ovary, the signals were observed in stage I, II and III oocytes (Figure 4D,E). Sense probes were also examined as negative control and no significant signals were detected (Figure 4C,F).

### 3.5. In Vitro RNAi-Mediated Cs-rchy1 Knockdown and Its Influence on the Expression of Sex-Related Genes

**Figure 5:**
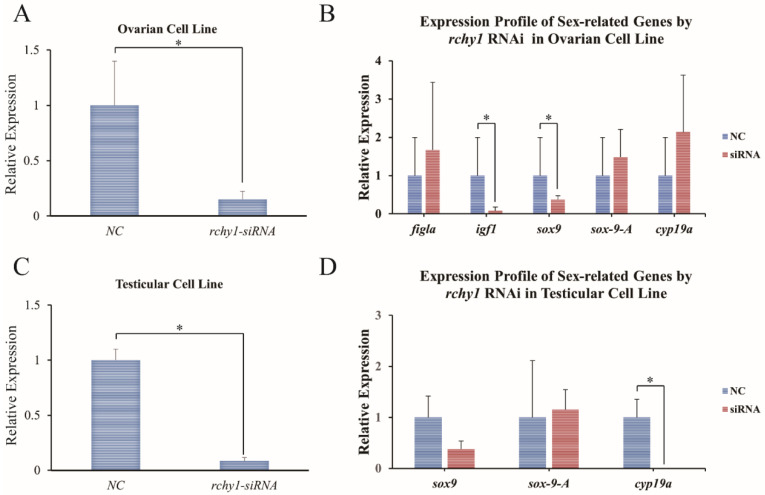
Relative mRNA expression levels of *Cs-rchy1*, *figla_tv1*, *igf1*, *sox9*, *sox-9-A*, and *cyp19a* in ovarian and testicular cell lines after RNAi treatment. (**A**) Expression of *Cs-rchy1* after the transfection of the siRNAs for 48 h in ovarian cell line. (**B**) Expression of *figla_tv1*, *igf1*, *sox9*, *sox-9-A*, and *cyp19a* after the transfection of the siRNAs for 48 h. (**C**) Expression of *Cs-rchy1* after the transfection of the siRNAs for 48h in testicular cell line. (**D**) Expression of *sox9*, *sox-9-A*, and *cyp19a* after the transfection of the siRNAs for 48 h. NC, negative control group. siRNA, *rchy1-siRNA* treated group. Asterisks (*) indicate significant differences (*p* < 0.05) between the treated group and the control.

The in vitro RNAi-mediated knockdown was performed using the Chinese tongue sole testicular and ovarian cell line. To determine the silencing effect, *Cs-rchy1* expression was examined by qPCR at 48 h after siRNA transfection. The expression was reduced approximately to 15% in ovarian cell line and 8.5% in testicular cell line compared with the control (Figure 5A,C). The mRNA level of *figla_tv1*, *igf1*, *sox9*, *sox-9-A*, *cyp19a* were also measured. As shown in Figure 5B, *igf1* and *sox9* were strongly reduced in ovarian cell line. In testicular cell line, *cyp19a* was significantly decreased compared to the control. The expression level of *sox9* was also lower after RNAi, although it was not significant. In addition, *figla_tv1* and *igf1* were not detected in the testicular cell line.

## 4. Discussion

The sex differentiation and gametogenesis in teleost is involved in many genes, such as male-biased gene *dmrt1*, *gsdf*, and female-biased gene *foxl2*, *cyp19a*, etc. [20,21,22,23] It is widely believed that ubiquitin pathway plays an important role in fish spermatogenesis. In *Solea senegalensis*, over 400 genes were identified to be involved in spermatogenesis, including ubiquitin activating enzymes E1 and ubiquitin hydrolase Uch [15]. In *Oncorhynchus mykiss*, protamine replacement, an important procedure in spermatogenesis, is strictly regulated by histone ubiquitination [13]. In *Cynoglossus semilaevis*, a Z-chromosome-linked ubiquitin ligase gene *neurl3* was suggested to be closely associated with spermatogenesis [24]. These findings imply that the ubiquitin pathway pose a regulatory role in teleost spermatogenesis. Based on our previous screening, another Z-chromosome-linked ubiquitin ligase gene *Cs-rchy1* has emerged due to its relatively high expression in the gonad compared to other tissues. However, unlike the testis-biased expression of *neurl3*, the expression level of *Cs-rchy1* was high both in testis and ovary, which attracted our attention to survey its potential role in spermatogenesis, or gametogenesis more exactly. In addition, it is also an interesting issue that whether ubiquitin pathway (or genes in this pathway) interacts with these identified sex-related genes and how it works?

During male gonadal development, *Cs*-*rchy1* showed a continuous increase in mRNA transcription and reached the peak level at 6 mph, consistent with the time of cellular differentiation in testis, featured as appearance of spermatocyte [25,26]. The expression subsequently reduced in mature testes while still maintained certain level [27,28]. In accordance with these data, ISH results exhibited relatively strong signals in germ cells, especially in spermatids and spermatozoa. The similar expression pattern and cellular localization to the previously reported *neurl3* suggest, *Cs-rchy1* may play potential role germ cell proliferation and maturation in spermatogenesis. In ovary, *Cs-rchy1* expression was rather stable along the different developmental stages, but it is worth noting that *Cs-rchy1* expression was higher at 1.5 and 3 ypf although not significantly. These stages represent mature stages, so *Cs-rchy1* might play a different role besides oogenesis and early gonadal development.

After the in vitro knockdown of *Cs*-*rchy1* in cultured testicular cells, *cyp19a* was significantly declined. *cyp19a* was an important molecule of the steroid hormone synthesis, and it was reported that *rchy1* acts as androgen receptor and participates in estrogen synthesis pathway [29,30]. Therefore, we speculated that *Cs*-*rchy1* was involved in testes differentiation and spermatogenesis by affecting steroid hormone synthesis. In ovarian cell line, *Cs*-*rchy1* knockdown seemed likely to significantly decrease in *sox9* but has no effect on *cyp19a*. As *sox9a* and *cyp19a* were reported to form a regulatory cascade, we postulated that *Cs-rchy1* was involved in ovarian development and oogenesis by regulating steroid hormone pathway [31]. Knockdown of *Cs*-*rchy1* also results in the down-regulated expression of the *igf1*, it is not surprising as *igf1* is frequently reported to function in reproduction [32,33]. However, IGF systems also functions in response to growth hormone stimulus and thus growth control [34]. Since one-year old tongue sole began to show growth discrepancy between male and female, together with the *Cs-rchy1* expression pattern (increased in ovary at mature stages), whether *Cs*-*rchy1* plays a potential role in growth requires further investigation. As the testicular and ovarian cell are predominantly somatic cells and the steroid concentration is not well determined, the RNA inference data might only represent the response at molecular level. It is definite that we should make effort to establish the gonadal stem cell line in future, which would be very helpful for the investigation of gene function in gonadal differentiation.

## 5. Conclusions

In this study, we have cloned and characterized the cDNA of a ubiquitin ligase gene *rchy1* in *C. semilaevis* (*Cs-rchy1*). *Cs-rchy1* was predominantly expressed in gonads compared to other tissues. Along the developmental stages, *Cs-rchy1* in testis exhibited a significant increase at 6 months post hatching, while the expression levels were rather stable in ovary. ISH results showed that *Cs-rchy1* was mainly expressed in germ cells (spermatids and spermatozoa in testis; stage I, II and III oocytes in ovary). In vitro RNAi found that *Cs-rchy1* knockdown resulted in the decline of *sox9* and *igf1* in the ovarian cell line and down-regulation of *cyp19a* in testicular cell line, suggesting the participation of *Cs-rchy1* in gonadal differentiation and gametogenesis in *C. semilaevis* via regulating steroid hormone synthesis, while its role in growth needs further investigation.

## Figures and Tables

**Table 1:** Primer sequences used in this study.

Primer	Sequences (5′–3′)	Purpose	Product Size
*rchy1* probeF	CACCGCGGCCGCAGCAATGGGAGCAGATAG	ISH	213 bp
*rchy1* probeR	CACCCTCGAGGCTGATGTTGTGCGTGAA		
*rchy1* F	TTTACACAAGACCTGCTTTG	qPCR	108 bp
*rchy1* R	TCATCTATCTGCTCCCATT		
*figla_tv1* F	ACATAGAGAAGTTCAAACGAGCC	qPCR	210 bp
*figla_tv1* R	CGGTAGCAGCTTTTAGTGTGTCT		
*sox9* F	AAGAACCACACAGATCAAGACAGA	qPCR	150 bp
*sox9* R	TAGTCATACTGTGCTCTGGTGATG		
*sox-9-A* F	GACCAAGTGTGTAATGTGACCAAG	qPCR	227 bp
*sox-9-A* R	GCTCTTGGTGTTGTTATATCCACG		
*cyp19a* F	GGTGAGGATGTGACCCAGTGT	qPCR	230 bp
*cyp19a* R	ACGGGCTGAAATCGCAAG		
*igf1* F	GTATCTCCTGTAGCCACACCCTCT	qPCR	137 bp
*igf1* R	GCCTCTCTCTCCACACACAAACT		
*β-actin* F	CCTTGGTATGGAGTCCTGTGGC	qPCR	150 bp
*β-actin* R	TCCTTCTGCATCCTGTCGGC		
*scaffold68-2F*	CCTAAATGATGGATGTAGATTCTGTC	Sex genotype	
*scaffold68-2R*	GATCCAGAGAAAATAAACCCAGG		

## Data Availability

The data presented in this study are available in this article.

## References

[1] Scheffner M., Nuber U., Huibregtse J.M. (1995). Protein ubiquitination involving an E1–E2–E3 enzyme ubiquitin thioester cascade. Nat. Cell Biol..

[2] Xu X.H., Fang W., Hao C., Wei S., Zhang X.S., Wang T. (2013). Transcript Profile Analyses of Maize Silks Reveal Effective Activation of Genes Involved in Microtubule-Based Movement, Ubiquitin-Dependent Protein Degradation, and Transport in the Pollination Process. PLoS ONE.

[3] Ohtake F., Baba A., Takada I., Okada M., Iwasaki K., Miki H., Takahashi S., Kouzmenko A., Nohara K., Chiba T. (2007). Dioxin receptor is a ligand-dependent E3 ubiquitin ligase. Nat. Cell Biol..

[4] Ehrlund A., Anthonisen E.H., Gustafsson N., Venteclef N., Remen K.R., Damdimopoulos A.E., Galeeva A., Pelto-Huikko M., Lalli E., Steffensen K. (2009). E3 Ubiquitin Ligase RNF31 Cooperates with DAX-1 in Transcriptional Repression of Steroidogenesis. Mol. Cell Biol..

[5] Rajapurohitam V., Bedard N., Wing S.S. (2002). Control of ubiquitination of proteins in rat tissues by ubiquitin conjugating enzymes and isopeptidases. Am. J. Physiol. Metab..

[6] An J.Y., Kim E.-A., Jiang Y., Zakrzewska A., Kim D.E., Lee M.J., Mook-Jung I., Zhang Y., Kwon Y.T. (2010). UBR2 mediates transcriptional silencing during spermatogenesis via histone ubiquitination. Proc. Natl. Acad. Sci. USA.

[7] Dickins R.A., Frew I., House C.M., O’Bryan M., Holloway A.J., Haviv I., Traficante N., de Kretser D.M., Bowtell D.D.L. (2002). The Ubiquitin Ligase Component Siah1a Is Required for Completion of Meiosis I in Male Mice. Mol. Cell Biol..

[8] Yin Y., Lin C., Kim S.T., Roig I., Chen H., Liu L., Veith G.M., Jin R.U., Keeney S., Jasin M. (2011). The E3 ubiquitin ligase Cullin 4A regulates meiotic progression in mouse spermatogenesis. Dev. Biol..

[9] Nian H., Zhang W., Shi H., Zhao Q., Xie Q., Liao S., Zhang Y., Zhang Z., Wang C., Han C. (2008). Mouse RING finger protein Rnf133 is a testis-specific endoplasmic reticulum-associated E3 ubiquitin ligase. Cell Res..

[10] Lerer-Goldshtein T., Bel S., Shpungin S., Pery E., Motro B., Goldstein R.S., Bar-Sheshet S.I., Breitbart H., Nir U. (2010). TMF/ARA160: A key regulator of sperm development. Dev. Biol..

[11] I Rodríguez C., Stewart C.L. (2007). Disruption of the ubiquitin ligase HERC4 causes defects in spermatozoon maturation and impaired fertility. Dev. Biol..

[12] Sun J., Shang X., Tian Y., Zhao W., He Y., Chen K., Cheng H., Zhou R. (2008). Ubiquitin C-terminal hydrolase-L1 (Uch-L1) correlates with gonadal transformation in the rice field eel. FEBS J..

[13] Nickel B.E., Roth S.Y., Cook R.G., Allis C.D., Davie J.R. (1987). Changes in the histone H2A variant H2A.Z and polyubiquitinated histone species in developing trout testis. Biochemistry.

[14] Redon E., Bosseboeuf A., Rocancourt C., Da Silva C., Wincker P., Mazan S., Sourdaine P. (2010). Stage-specific gene expression during spermatogenesis in the dogfish (Scyliorhinus canicula). Reproduction.

[15] Forne I., Castellana B., Marín-Juez R., Cerda J., Abián J., Planas J.V. (2011). Transcriptional and proteomic profiling of flatfish (Solea senegalensis) spermatogenesis. Proteomics.

[16] Liu Y., Chen S., Gao F., Meng L., Hu Q., Song W., Shao C., Lv W. (2017). SCAR transformation of sex-specific SSR marker and its application in half-smooth tongue sole (*Cynoglossus semiliaevis*). J. Agric. Biotechnol..

[17] Fryburg D.A. (1994). Insulin-like growth factor I exerts growth hormone- and insulin-like actions on human muscle protein metabolism. Am. J. Physiol. Metab..

[18] Zhang B., Wang X., Sha Z., Yang C., Liu S., Wang N., Chen S.-L. (2011). Establishment and Characterization of a Testicular Cell Line from the Half-Smooth Tongue Sole, *Cynoglossus semilaevis*. Int. J. Biol. Sci..

[19] Sun A., Wang T.Z., Wang N., Liu X.F., Sha Z.X., Chen S.L. (2015). Establishment and characterization of an ovarian cell line from half-smooth tongue sole *Cynoglossus semilaevis*. J. Fish Biol..

[20] Cui Z., Liu Y., Wang W., Wang Q., Zhang N., Lin F., Wang N., Shao C., Dong Z., Li Y. (2017). Genome editing reveals dmrt1 as an essential male sex-determining gene in Chinese tongue sole (*Cynoglossus semilaevis*). Sci. Rep..

[21] Zhu Y., Meng L., Xu W., Cui Z., Zhang N., Guo H., Wang N., Shao C., Chen S. (2018). The autosomal Gsdf gene plays a role in male gonad development in Chinese tongue sole (*Cynoglossus semilaevis*). Sci. Rep..

[22] Dong X., Chen S., Ji X., Shao C. (2011). Molecular cloning, characterization and expression analysis of Sox9a and Foxl2 genes in half-smooth tongue sole (*Cynoglossus semilaevis*). Acta Oceanol. Sin..

[23] Deng S.-P., Chen S.-L., Xu J.-Y., Liu B.-W. (2009). Molecular cloning, characterization and expression analysis of gonadal P450 aromatase in the half-smooth tongue-sole, *Cynoglossus semilaevis*. Aquaculture.

[24] Xu W., Li H., Dong Z., Cui Z., Zhang N., Meng L., Zhu Y., Liu Y., Li Y., Guo H. (2016). Ubiquitin ligase gene neurl3 plays a role in spermatogenesis of half-smooth tongue sole (*Cynoglossus semilaevis*) by regulating testis protein ubiquitination. Gene.

[25] Chen S., Zhang G., Shao C., Huang Q., Liu G., Zhang P., Song W., An N., Chalopin D., Volff J.-N. (2014). Whole-genome sequence of a flatfish provides insights into ZW sex chromosome evolution and adaptation to a benthic lifestyle. Nat. Genet..

[26] Li H., Xu W., Zhu Y., Zhang N., Ma J., Sun A., Cui Z., Gao F., Wang N., Shao C. (2017). Characterization and expression pattern of r-spondin1 in *Cynoglossus semilaevis*. J. Exp. Zool. Part B Mol. Dev. Evol..

[27] Chen S.-L., Tian Y.-S., Yang J.-F., Shao C., Ji X.-S., Zhai J.-M., Liao X.-L., Zhuang Z.-M., Su P.-Z., Xu J.-Y. (2008). Artificial Gynogenesis and Sex Determination in Half-Smooth Tongue Sole (*Cynoglossus semilaevis*). Mar. Biotechnol..

[28] Liu Y., Zhang W., Du X., Zhao J., Liu X., Li X., Zhang Q., Wang X. (2017). Sexually dimorphic expression in developing and adult gonads shows an important role of gonadal soma-derived factor during sex differentiation in olive flounder ( Paralichthys olivaceus ). Comp. Biochem. Physiol. Part B Biochem. Mol. Biol..

[29] Beitel L.K., A Elhaji Y., Lumbroso R., Wing S.S., Panet-Raymond V., Gottlieb B., Pinsky L., A Trifiro M. (2002). Cloning and characterization of an androgen receptor N-terminal-interacting protein with ubiquitin-protein ligase activity. J. Mol. Endocrinol..

[30] Morgan C. Micro RNAs and the Sex Specific Development of the Neonatal Brain: A Point of Vulnerability to the Programming Effects of Prenatal Stress. **2015**. Publicly Accessible Penn Dissertations. 1098. https://repository.upenn.edu/edissertations/1098.

[31] Huang X., Qin Q., Gong K., Wu C., Zhou Y., Chen Q., Feng W., Xing Y., Wang C., Wang Y. (2020). Comparative analyses of the Sox9a-Amh-Cyp19a1a regulatory Cascade in Autotetraploid fish and its diploid parent. BMC Genet..

[32] Lucy M.C. (2012). Growth hormone regulation of follicular growth. Reprod. Fertil. Dev..

[33] Reinecke M. (2010). Insulin-like Growth Factors and Fish Reproduction. Biol. Reprod..

[34] Puche J.E., Castilla-Cortázar I. (2012). Human conditions of insulin-like growth factor-I (IGF-I) deficiency. J. Transl. Med..

